# Elucidating the network features and evolutionary attributes of intra- and interspecific protein–protein interactions between human and pathogenic bacteria

**DOI:** 10.1038/s41598-020-80549-x

**Published:** 2021-01-08

**Authors:** Debarun Acharya, Tapan K. Dutta

**Affiliations:** Department of Microbiology, Bose Institute, P-1/12, CIT Scheme VII M, Kolkata, West Bengal 700 054 India

**Keywords:** Computational biology and bioinformatics, Evolution, Systems biology

## Abstract

Host–pathogen interaction is one of the most powerful determinants involved in coevolutionary processes covering a broad range of biological phenomena at molecular, cellular, organismal and/or population level. The present study explored host–pathogen interaction from the perspective of human–bacteria protein–protein interaction based on large-scale interspecific and intraspecific interactome data for human and three pathogenic bacterial species, *Bacillus anthracis, Francisella tularensis* and *Yersinia pestis*. The network features revealed a preferential enrichment of intraspecific hubs and bottlenecks for both human and bacterial pathogens in the interspecific human–bacteria interaction. Analyses unveiled that these bacterial pathogens interact mostly with human party-hubs that may enable them to affect desired functional modules, leading to pathogenesis. Structural features of pathogen-interacting human proteins indicated an abundance of protein domains, providing opportunities for interspecific domain-domain interactions. Moreover, these interactions do not always occur with high-affinity, as we observed that bacteria-interacting human proteins are rich in protein-disorder content, which correlates positively with the number of interacting pathogen proteins, facilitating low-affinity interspecific interactions. Furthermore, functional analyses of pathogen-interacting human proteins revealed an enrichment in regulation of processes like metabolism, immune system, cellular localization and transport apart from divulging functional competence to bind enzyme/protein, nucleic acids and cell adhesion molecules, necessary for host-microbial cross-talk.

## Introduction

Pathogen–host interaction is the perfect example of evolutionary arms race where sustained coevolution is continuously shaping the hosts’ and pathogens’ genome and life history characteristics. The success and failure of the development of a disease depend on the survival, reproduction, and transmission of a pathogen into a host, which is countered by the host-resistance and immune system components.

Pathogen–host interactions are better understood from molecular perspectives, where pathogens hijack and manipulate the host’s cellular machinery and immune system components for their growth, thereby establishing a pathogen-host protein–protein interaction (PHPPI) network inside a host^1^. In plants, such interactions are mediated by pathogen effectors, which are pathogen proteins, translocated inside host cells and target particular host genes/proteins to interfere with host cellular mechanisms, eventually causing infections^2^. In human-pathogen interaction, proteins from both human and pathogen are involved in the PHPPI network that ultimately leads to either disease progression or elimination of pathogen from the human body. The human protein–protein interaction represents a scale-free distribution, where the majority of the proteins interact with only a few proteins while there are a few proteins that interact with a large number of proteins. Such a distribution increases the robustness of the human PPI network against random pathogen attacks. Therefore, in order to cause pathogenicity, pathogens target particular human proteins (directed attack) for their growth and establishment^3^. Conversely, the strategy of the human cellular system is to resist the pathogen attack by hindering its growth and ultimately eliminating it, which is mostly mediated by the human immune system components^4,5^. Pathogens that evade the immune system can be killed by targeted therapeutics like broad-spectrum or specific antibiotics. However, with the increasing ability of pathogens to evade both the human immune system and antibiotics^6^, it has become more difficult to counter such infectious agents. The human–pathogen interactome is now considered very important for studying pathogenic disease, as it provides crucial information on the virulence factors along with their interactions essential for pathogenicity at the system level^1,7^. The accumulation of PHPPI data in the last decade paved the way for system-level analyses with the whole interactome, leading to a better understanding of the pathogenicity, disease progression, and human–pathogen coevolution for a better therapeutic approach to prevent and cure infections.

A detailed analysis of interspecific pathogen–human protein–protein interaction revealed that pathogen proteins mainly interact with proteins having high centrality values in the human PPI network. This includes hubs and bottlenecks, proteins having a high degree and betweenness centrality, respectively^2,8,9^. Although both these groups of proteins are functionally important counterparts of the human PPI network, often essential for host survival^10,11^, a phenomenon known as “centrality-lethality rule”^12,13^, the group that interacts more with pathogen proteins are not known. Additionally, these proteins evolve at a slower rate^14–17^, providing an opportunity for a sustainable host–pathogen interaction for over a long evolutionary time scale, a beneficial event for pathogen species. Moreover, higher connectivity of these pathogen-interacting hub proteins may bring about an increased influence of pathogen protein on the components of the human PPI network. The hubs with their interacting partners, form functional modules, each assigned to a specific function, where they may either act as intramodular or party-hubs (participating in the same functional module with their interacting partners) or intermodular or date-hubs (participating in different functional module with different interacting partners). However, it will be interesting to know which of these hubs interact more often with pathogen proteins, as it can be useful to understand the functional modules that get targeted by the pathogens for their pathogenicity and disease progression.

Most of the human–pathogen interactions are focused on viral infections, where viruses hijack the human transcriptional machinery to synthesize their proteins. The viral proteins evolve in a very sophisticated manner, and their interactions with human proteins often involve short linear motifs (SLiMs) present in the latter^18,19^. However, the interspecific PPI data between human and a majority of bacterial pathogens are not comprehensive. Thus, very little is known on human bacteria protein–protein interaction where pathogenic bacteria also interacts with human hubs and hijack the immune system components to evade host immune response^1^. In the present study, we explored the attributes of human bacteria protein–protein interaction from three pathogenic bacterial species, *Bacillus anthracis*, *Francisella tularensis,* and *Yersinia pestis* for which large-scale interspecific PPI data is available. All these pathogenic bacteria are enlisted as ‘Category A bioterrorism agents’. In addition, in silico approaches were undertaken to understand various aspects of the human–bacteria protein–protein interaction network and its participants, to better understand the mechanism of pathogenicity and disease progression.

## Results and discussion

### Hubs and Bottlenecks in pathogen-interacting and non-interacting human proteins

**Figure 1:**
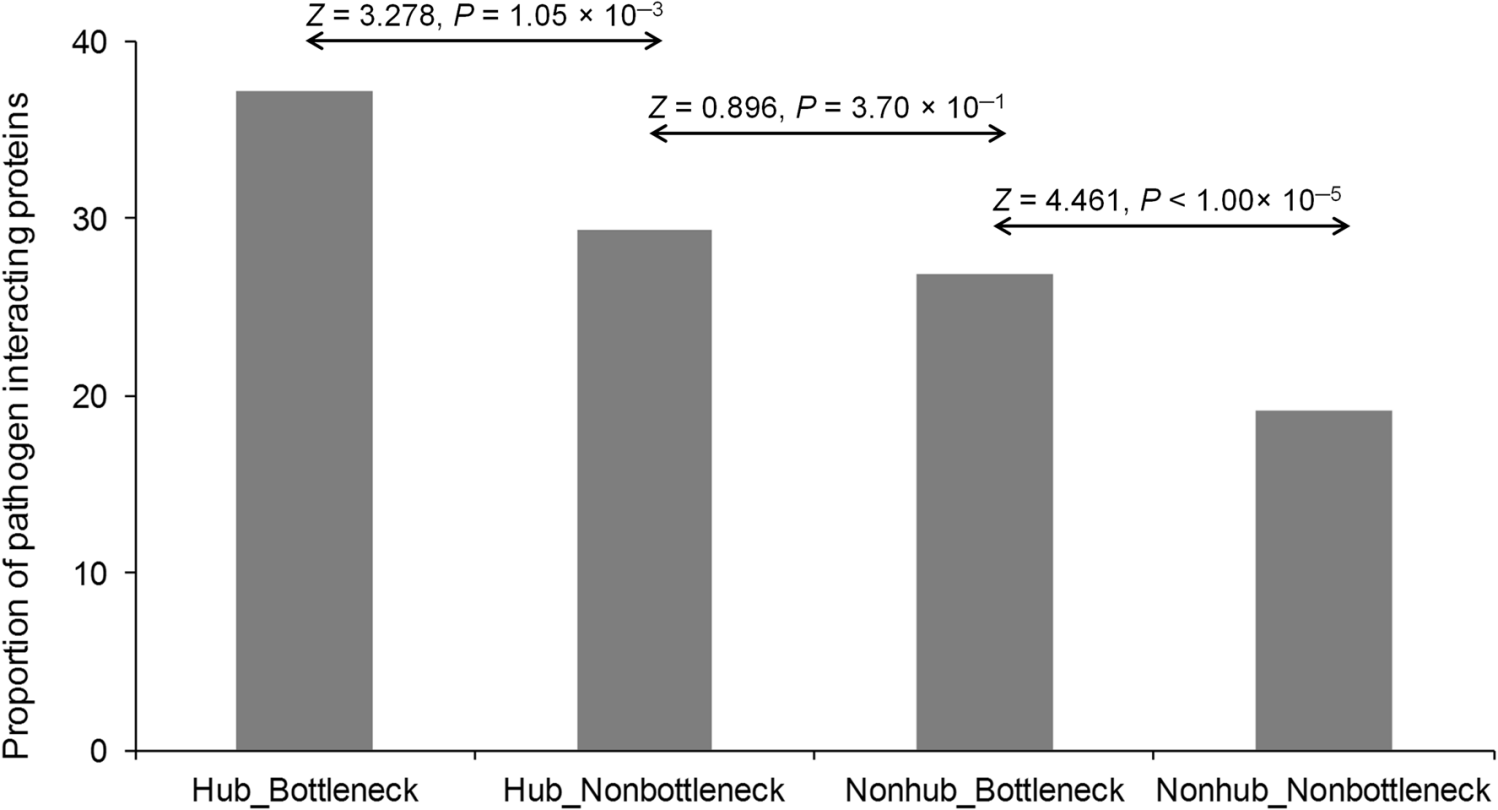
Proportion of pathogen interacting proteins in human hub-bottleneck, hub-nonbottleneck, nonhub-bottleneck and nonhub-nonbottleneck proteins.

The human–bacteria protein–protein interaction networks for three bacterial pathogens, namely *Bacillus anthracis*, *Francisella tularensis,* and *Yersinia pestis* were analyzed to understand the network features of bacterial protein-interacting human proteins. In general, the protein–protein interaction (PPI) data contains many false positives and false negatives. Here, we selected three bacterial species for this study that have the highest number of interspecific interactions with human proteins verified by multiple databases. Additionally, the PPI data is not yet comprehensive and therefore, all the interpretations are made from the currently available data. It has been previously reported that the pathogen proteins mainly interact with the highly connected host proteins (host-hubs)^1,20^. In this study, we classified the human proteins into four groups: (a) not-interacting with any bacterial pathogen, (b) interacting with only one pathogen, (c) interacting with only two pathogens and (d) interacting with all three pathogens. The human protein–protein interaction network was constructed using the PICKLE database, where the PPIs supported by any two of four widely used PPI databases (BIOGRID^21^, MINT^22^, HPRD^23^, DIP^24^ and IntAct^25^) were considered as true-interaction. The final data contain 11,815 proteins involved in 61,273 high-quality interactions, representing a little less than half of the human proteome. Comparing the proportion of hubs, it has been observed that the pathogen-interacting human proteins correspond to a higher proportion of hubs and bottlenecks than that of the non-interacting group (Supplementary Table S3). The pathogen-interacting proteins also have higher mean interacting partners (degree centrality) than that of the non-interacting group with respect to both hubs and nonhubs. Additionally, human proteins that interact with more bacterial pathogens have a higher proportion of hubs and higher mean interacting partners than those interacting with fewer pathogens (Table 1). This suggests that pathogenic proteins preferentially target human hubs and bottlenecks that comprise functionally most important proteins in the human protein interaction network, which in turn, may damage the functional implication of the network. The high degree centrality of pathogen-interacting human proteins may also ensure the pathogens’ establishment within the human host via its control over a broad range of target human proteins. When human proteins were classified into hub-bottlenecks, hub-nonbottleneck, nonhub-bottleneck, and nonhub-nonbottleneck based on these two centrality measures, the highest proportion of pathogen-interacting proteins was obtained in the hub-bottleneck class. More interestingly, the hub-nonbottleneck and nonhub-bottleneck possess no significant difference, which indicates that hubs and bottlenecks are equally targeted by proteins of these pathogens (Fig. 1).

**Table 1:** Proportion of hubs and bottlenecks in human proteins based on their interactions with bacterial pathogens.

Pathogen interaction status	Total proteins	Hubs	% Hubs	Mean interacting partners	Significance
**Proportion of Hubs in pathogen-interacting and non-interacting human proteins**					
Non-interacting	9137	1498	16.39	8.95	P_%Hubs_ = 6.12 × 10^─65^, Fisher’s exact test; P_Mean_Interaction_Partner_ = 5.09 × 10^─92^, Kruskal–Wallis test
Interacting with one	1802	468	25.97	12.82	
Interacting with two	635	250	39.37	18.07	
Interacting with three	241	101	41.91	23.84	

Pathogen interaction status	Total proteins	Bottlenecks	%Bottlenecks	Significance	
**Proportion of Bottlenecks in pathogen-interacting and non-interacting human proteins**					
Non-interacting	9137	1540	16.85	P_%Bottleneck_ = 1.96 × 10^─58^, Fisher’s exact test	
Interacting with one	1802	477	26.47		
Interacting with two	635	235	37.01		
Interacting with three	241	105	43.57

Moreover, the whole protein interaction network can be subdivided into many functional modules, with each distinct module representing a specific function. Based on modularity, the hubs which belong to the same functional module as their interacting partners are known as intramodular hubs or party hubs, and those having interacting partners that belong to different functional modules are known as intermodular hubs or date hubs. To evaluate the preferential interaction of pathogen proteins with any one class of these hubs, the human party- and date hubs were identified using co-expression values of human proteins and their interacting partners and their interacting interface (see “Materials and methods”). Based on the above, the proportion of party hubs was found to be significantly higher in pathogen-interacting proteins, signifying pathogen proteins target some of the functional modules for their benefit (Table 2).

**Table 2:** Proportion of party-hubs and date-hubs in pathogenic bacteria-interacting and non-interacting human proteins.

Pathogen-interaction status	Total hubs	Party hubs	%Party hubs	Date hubs	%Date hubs	Statistical significance
**Using PCC > 0.5 to determine party hubs**						
Interacting	802	123	15.34	679	84.66	Z = 3.965 P = 7.30 × 10^─5^
Noninteracting	1441	140	9.72	1301	90.28	
**Using PCC > PCC**_**mean**_** to determine party hubs**						
Interacting	802	503	62.72	299	37.28	Z = 5.035 P < 1.00 × 10^─5^
Noninteracting	1441	745	51.70	696	48.30	

### Hubs and Bottlenecks in human-interacting and non-interacting bacterial proteins

The scale-free network topology follows power-law node degree distribution, comprising a few nodes with a higher degree centrality than many other nodes. Such a network is resilient against random-attacks, which applies to human as well as pathogenic bacteria alike (Supplementary Fig. 1). In order to disrupt the human PPI network, the pathogen proteins need to act against particular human proteins via non-random directed interactions. The pathogenic proteins with high degree centrality may be potential candidates involved in such disruption, due to their inherent property of high interaction ability. To explore this further, we subdivided the pathogen proteins into hubs or nonhubs based on their degree centrality and bottlenecks or nonbottlenecks, based on betweenness centrality (see “Materials and methods”). Following this classification, the network properties of human-interacting and non-interacting pathogen proteins were explored and it was observed that the bacterial proteins which interact with human proteins are significantly enriched in bacterial hubs and bottlenecks in the bacterial PPI network. These hub proteins also have higher mean interacting partners (Table 3), indicating that the human-interacting pathogen proteins have the potential to interact with multiple type of proteins in the intraspecific PPI network, which may facilitate in interspecific host–pathogen interactions.

**Table 3:** Proportion of hubs and bottlenecks in bacterial pathogens’ PPI network in human-interacting and non-interacting proteins.

Species	Protein class/human-interacting?	Total proteins	Hubs	%Hubs	Bottlenecks	%BNs	Significance (%hubs, %BNs)
*Bacillus anthracis* (N = 5089)	Interacting	316	90	28.48	97	30.70	Z_%hub_ = 3.922, Z_%BNs_ = 4.990 P < 0.001
	Noninteracting	2966	569	19.18	560	18.88	
*Francisella tularensis *(N = 1485)	Interacting	296	73	24.66	79	26.69	Z_%hub_ = 2.192, Z_%BNs_ = 3.292 P < 0.05
	Noninteracting	870	163	18.74	155	17.82	
*Yersinia pestis *(N = 3893)	Interacting	340	89	26.18	96	28.24	Z_%hub_ = 3.009, Z_%BNs_ = 4.000 P < 0.01
	Noninteracting	2529	486	19.22	480	18.98	

### Gene essentiality of pathogen-interacting human proteins

**Figure 2:**
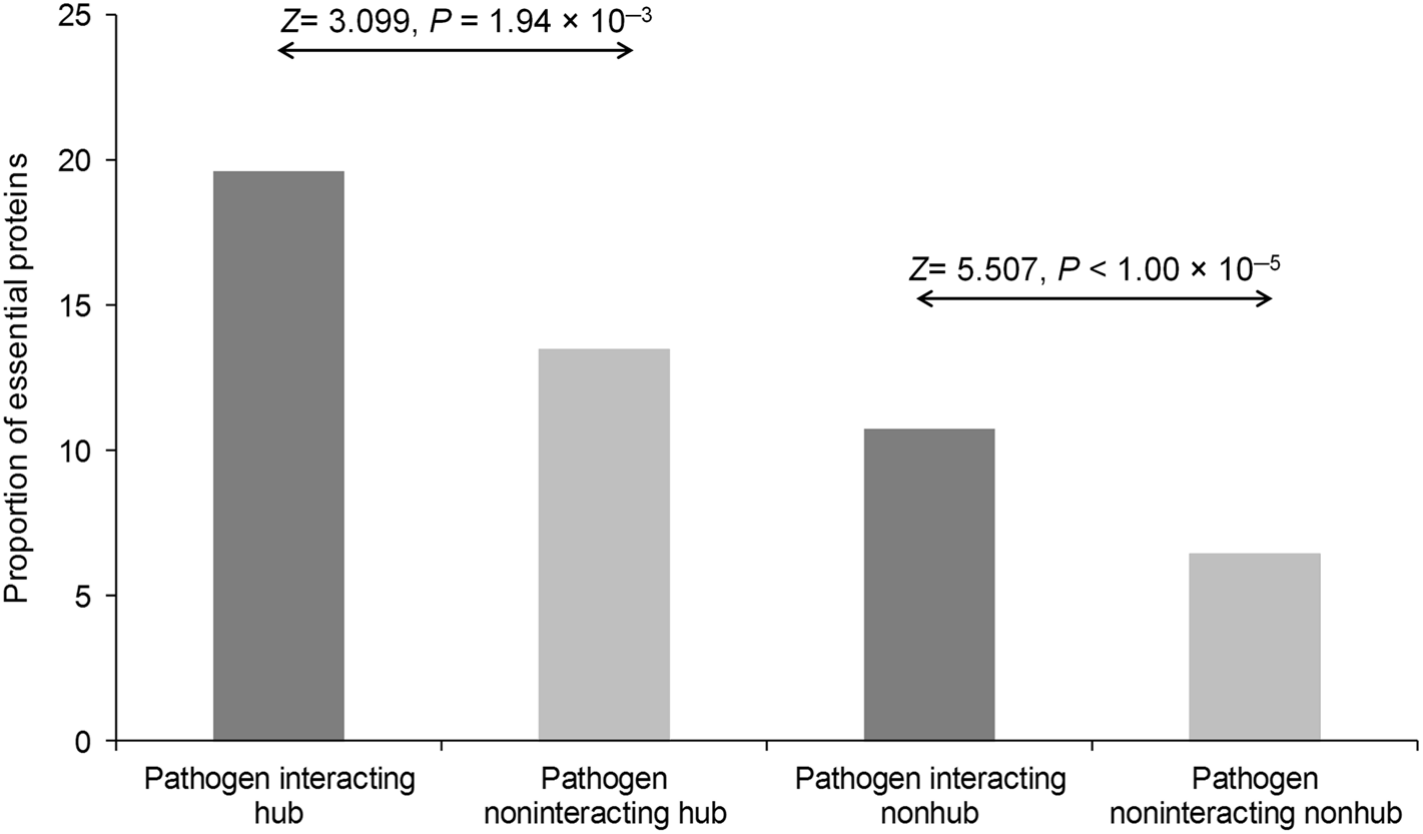
Proportion of essential proteins in pathogen-interacting and noninteracting hubs and nonhubs.

Genes indispensable to the survival and reproduction of an organism are considered as essential genes^26,27^. Proteins encoded by such genes are associated with vital molecular functions and are under strong purifying selection. It had been observed that the pathogen-interacting proteins comprise a higher proportion of essential proteins, which however, maybe due to their enrichment among hubs^10,28^. Moreover, when we considered hub and nonhub proteins separately, the pathogen-interacting proteins were found to be enriched in essential proteins for both groups, suggesting that these deadly pathogens may disrupt vital functions of the host, thereby facilitating pathogenicity and disease progression (Fig. 2).

### Evolutionary rates of pathogen-interacting and noninteracting human proteins

**Figure 3:**
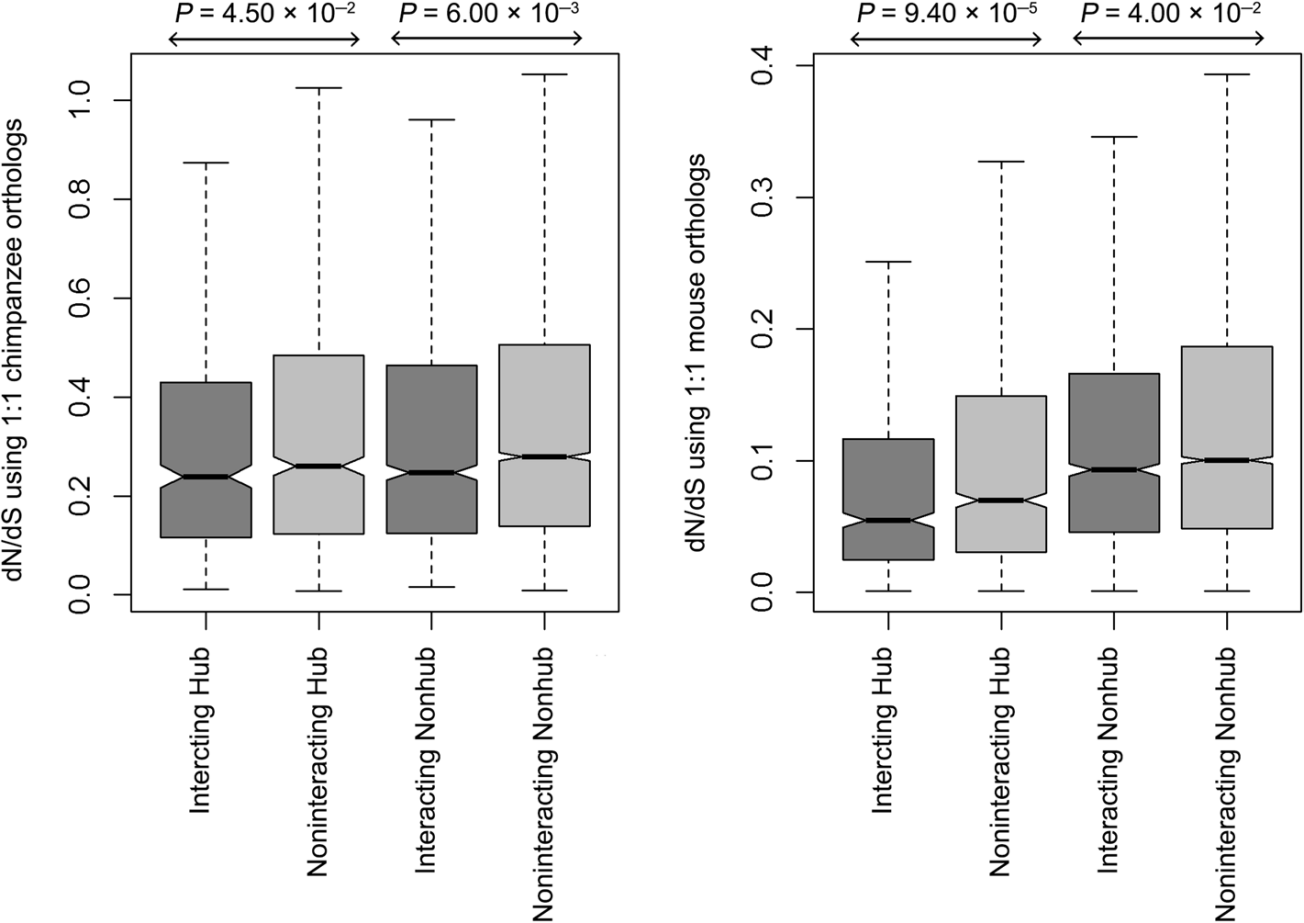
Evolutionary rate (dN/dS ratio) of pathogen-interacting and noninteracting human proteins within hubs and nonhubs using 1:1 chimpanzee and mouse orthologs.

The evolutionary rate of proteins depicts the change in its amino acid sequence over time. As hubs are evolutionarily more conserved than nonhubs and also enriched with pathogen-interacting proteins, they are supposed to reveal a slower evolutionary rate. However, very little is known regarding the differences in evolutionary rate between pathogen-interacting and noninteracting hubs. Considering pathogen-interacting/-noninteracting hubs/nonhubs, a comparison of the evolutionary rate as dN/dS ratio using 1:1 Mouse and Chimpanzee orthologs^29^ revealed a slower evolutionary rate in hub proteins. Nevertheless, among the pathogen-interacting and noninteracting hubs, the former shows a slower evolutionary rate (Fig. 3), suggesting that the evolutionarily more conserved hubs are more likely to be targeted by pathogens. It is, however, beneficial from the pathogens’ perspective, as it may allow an efficient pathogen–host protein–protein interaction throughout large evolutionary time-scale.

### Intrinsic disorder of pathogen-interacting and noninteracting human proteins

Functional implication of protein is always mediated by its proper three-dimensional configuration. However, there are certain amino acid residues or stretches in proteins’ sequence, which do not let a protein fold into a definite conformation, and under such a situation, its associated flexibilities often facilitate in imparting productive protein–protein interactions. Such residues/regions on a protein are known as intrinsically disordered residues/regions. Intrinsically disordered proteins, naturally, lack distinct three-dimensional structure but can adopt definite conformation upon their interaction with other proteins, facilitating low-affinity interactions with high-specificity^30^. Proteins that are highly connected in a network of proteins are usually rich in these regions^31^, which may play an important role in the interactions between host and pathogen proteins. Although bacterial proteins are less disordered than the human proteins^32,33^, the disordered regions in human proteins are supposed to be utilized by the bacterial pathogens as potential regions for interaction. To address the same, IUPred algorithm was used to identify the disordered residues in pathogen-interacting and non-interacting proteins^34^. The proportion of disordered proteins (P_disordered_) in the pathogen-interacting proteins is significantly higher than the non-interacting proteins (P_disordered_interacting_ = 59.73, N_interacting_ = 2677, P_disordered_noninteracting_ = 49.07, N_noninteracting_ = 9136, Z = 9.706, P < 1.00 × 10^−4^), suggesting that they may play an important role in pathogen–host interactions. Additionally, when the total number and percentage of disordered regions and residues of individual proteins were considered, we found that pathogen interacting proteins have a higher number and mean percentage of long disordered regions and disordered residues (Supplementary Table S4), indicating human proteins with intrinsically disordered regions and residues are more prone to pathogen-attack. However, as smaller disordered segments can also be important for interaction, therefore we also considered the proteins having ≥ 15 residue long disordered stretches, which gives a consistent result (Supplementary Table S4).

**Figure 4:**
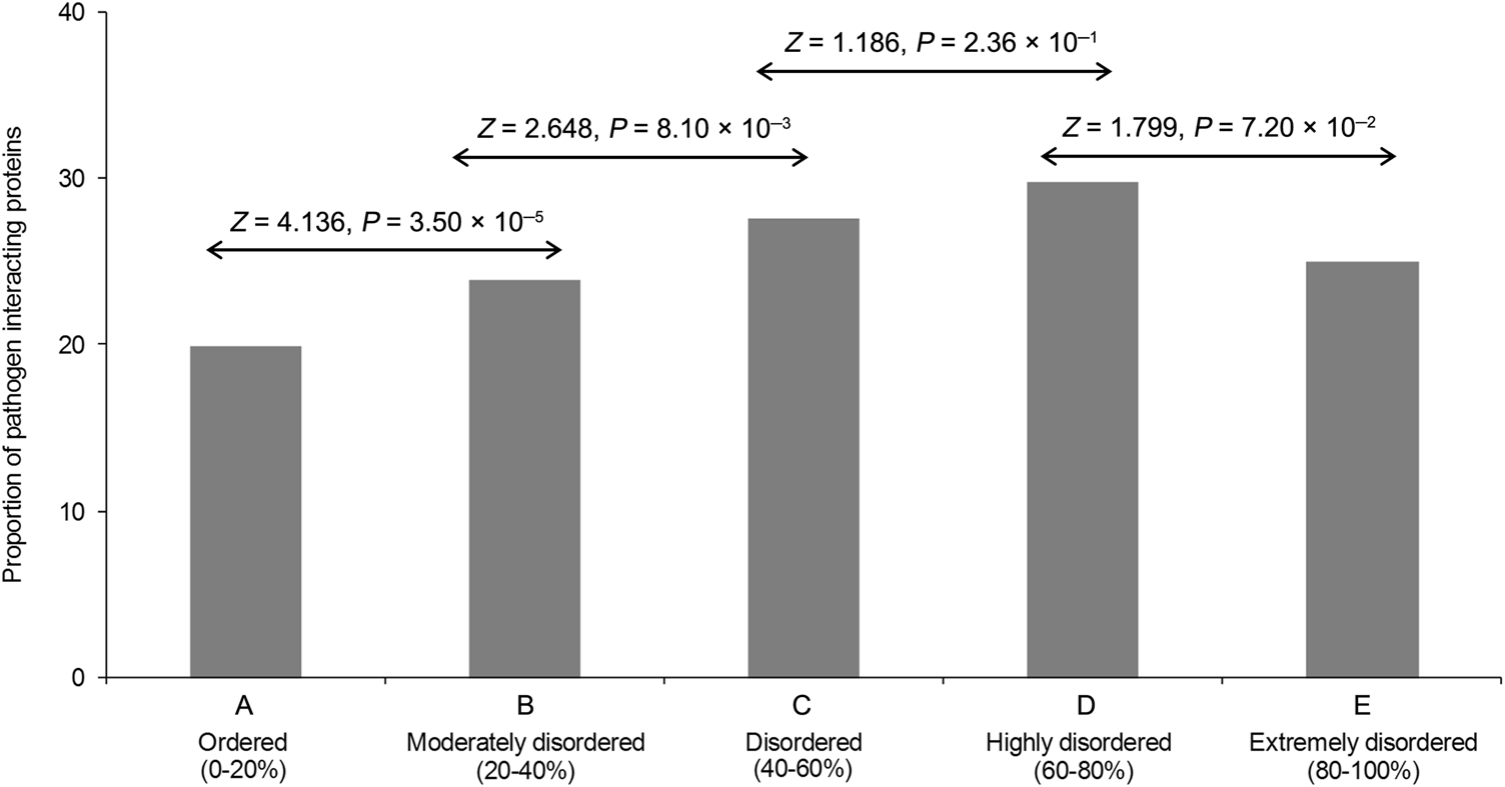
Proportion of pathogen-interacting human proteins belonging to different disorder bins.

To further strengthen the claim as stated above, the number of interacting pathogen proteins for each of the three bacteria were calculated for each human protein and it appears to hold a significant positive correlation with the amount of disorder content present in the human protein (Supplementary Table S5). When the human proteins were binned based on their disorder content into five bins (see “Materials and methods”), it was observed that the proportion of pathogen-interacting genes increases gradually with increasing disorder content up to 80% (Fig. 4). Together, these results suggest that the protein intrinsic disorder plays a major role in the host–pathogen interactions.

### Molecular recognition features (MoRFs) in pathogen-interacting and noninteracting human disordered proteins

We also considered the Molecular Recognition Features or MoRFs, which are 5–25 residues long specialized elements located within the disordered regions of proteins that undergo disorder to order transition upon binding with their respective interacting partners. Here, to understand whether the disordered regions in pathogen interacting human proteins can serve as the disordered protein binding sites for pathogen proteins, we explored the MoRFs within the human disordered proteins, using the fMoRFpred^35^ webserver. The pathogen interacting human proteins were found to be rich in molecular recognition features (MoRFs) than the noninteracting counterpart (MoRF_regions_interacting_ = 1.017, MoRF_regions_noninteracting_ = 0.931, P = 3.949 × 10^−2^ ; MoRF_residues_interacting_ = 15.035, MoRF_residues_noninteracting_ = 12.765, P = 3.718 × 10^−9^, Mann–Whitney U test, N_interacting_ = 1599, N_noninteracting_ = 4472), suggesting that pathogen-interacting human proteins are more enriched in these regions, which may favour the interspecific protein–protein interaction.

### Protein domains in pathogen-interacting and non-interacting human proteins

**Figure 5:**
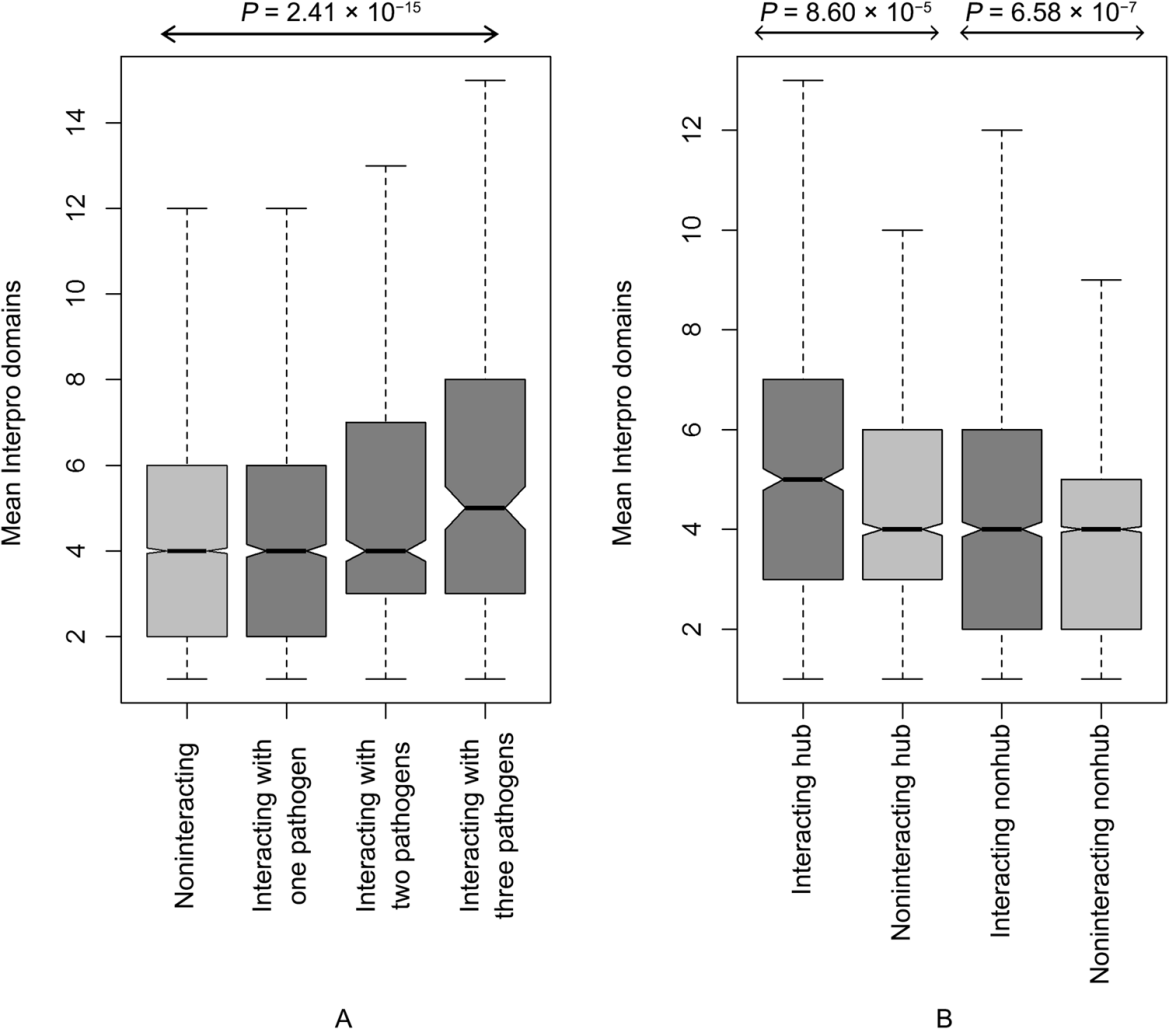
Mean Interpro protein domains: (**A**) pathogen noninteracting and interacting human proteins subdivided n number of interacting pathogens for a particular human protein; (**B**) pathogen interacting and noninteracting human proteins subdivided in hub and nonhub classes.

Although, protein intrinsic disorder facilitates protein–protein interaction by providing flexibility to the proteins’ structure^36^, protein domains, the most conserved and functionally essential part of a protein serve a distinct role in such interaction^37^. More specifically, the protein–protein interaction can be viewed as interaction between domains of different proteins. Therefore, proteins with a greater number of domains may have a higher probability of interaction with other proteins. To study the influence of protein domains on human-bacteria interaction, the mean number of domains of pathogenic bacteria interacting- and noninteracting-human proteins were calculated using Interpro repository^38^. It was observed that the pathogen-interacting proteins contain a higher number of domains than that of the noninteracting ones (P = 6.73 × 10^−16^, Mann–Whitney U test). Moreover, the higher number of domains in pathogen-interacting human proteins may be attributable to the abundance of hubs within them. Thus, we divided the data into hubs and nonhubs. Interestingly, within both hubs and nonhubs, the pathogen-interacting proteome has a higher number of domains (P_hub_ = 8.60 × 10^−5^, P_nonhub_ = 6.58 × 10^−7^). Additionally, the proteins interacting with more pathogens hold a higher number of protein domains (P = 2.41 × 10^−15^, Kruskal–Wallis test) (Fig. 5). This suggests that proteins with a higher domain number have a higher probability of interaction with pathogen proteins, facilitated via interspecific domain–domain interaction.

### Functional enrichment analysis of pathogen-interacting proteins

The association of party hubs with pathogen proteins indicates that these bacterial pathogens mostly target particular functional modules of human proteome for the establishment of pathogenicity and progression of the disease. For a detailed insight, the functional enrichment of the pathogen-interacting human proteins was studied using the Humanmine^39^ and Gorilla^40^ webservers. The top 10 enriched Gene Ontology (GO) terms matched in both the datasets were observed for both the GO domains, ‘Biological Process’ and ‘Molecular Function’ (Supplementary Table S6). The pathogen-interacting proteins were revealed to be enriched in processes like regulation of biological/cellular processes, cellular localization, immune system, interspecies interaction between organisms, regulation of cellular (metabolic) processes, regulation of nitrogen compound metabolic processes, regulation of primary metabolic processes, and vesicle-mediated transport processes. These proteins were also shown to be enriched in functions like RNA binding, enzyme/protein binding, nucleic acid binding, protein-containing complex biomolecule binding, cadherin binding, cell adhesion molecule binding, transcription factor binding, chromatin binding, and kinase binding. The above functional enrichment clearly suggest that during pathogenesis, these pathogens primarily regulate the processes related to immune system, cellular localization and transport, apart from influencing the binding of host macromolecules and cell-adhesion molecules, necessary for host-microbial cross-talks.

## Materials and methods

### Protein–protein interaction datasets

The human–bacteria protein-interaction data for the three bacterial species namely *Bacillus anthracis*, *Francisella tularensis,* and *Yersinia pestis* were obtained from four well established host–pathogen interactome databases: APID (Agile Protein Interactome Dataserver, http://cicblade.dep.usal.es:8080/APID/init.action#tabr1^41^; MENTHA, https://mentha.uniroma2.it/^42^, HPI-DB (Host Pathogen Interaction Database), http://hpidb.igbb.msstate.edu/index.html^43^ and PHISTO (Pathogen Host Interaction Search Tool), http://www.phisto.org/browse.xhtml^44^. The binary interactions reported in no less than three of the four databases were used in this study as the pathogen-interacting human proteins. The human proteins and their sequences were obtained from Uniprot (https://www.uniprot.org/)^45^. The human proteins with no reported interaction with none of the pathogen protein in either of the databases were considered as pathogen-non-interacting proteins (Supplementary Table S1).

The human PPI data was obtained from PICKLE (Protein InteraCtion KnowLedgebasE) (www.pickle.gr)^45^, which combines all the globally used protein–protein interaction database like BIOGRID^21^, MINT^22^, HPRD^23^, DIP^24^ and IntAct^25^. We removed all the self-interactions and considered interactions supported by at least two of these databases for our study^45^.

The within-species PPI data of all three bacterial pathogens were obtained from the STRING database (https://string-db.org/)^46^, considering the experimentally validated interactions only. The STRING IDs were annotated to Uniprot IDs using the annotation file present in the STRING database. Reciprocal BLAST with 100% sequence identity and e-values < e^−10^ BLAST parameters was used to determine the orthologous proteins of two different pathogen strains belonging to the same species as available in pathogen–PPI and pathogen–human PPI databases. The final dataset consists of 122,546 *Homo sapiens* binary interactions involving 11,833 proteins, 277,210 *B. anthracis* binary interactions involving 3285 proteins, 53,614 *F. tularensis* interactions involving 1167 proteins and 135,090 *Y. pestis* binary interactions involving 2872 proteins. We analyzed each network using the Network Analyzer plugin of Cytoscape (version 3.7.1) to get the degree and betweenness centrality. The node degree of all the species shows power-law distributions (Supplementary Fig. S1). We subdivided the proteins of each species into hubs and nonhubs depending on their degree centrality. The top ~ 20% proteins of the node degree distribution having the highest number of interacting partners were considered as hubs, while the rest as nonhubs, according to the 20–80 rule of power-law distributions (Pareto principle)^47^. Similarly, we classified the proteins into bottlenecks (proteins that are central to many paths in the network) and non-bottlenecks considering the proteins representing the top ~ 20% of betweenness centrality as bottlenecks and the rest as non-bottlenecks (Supplementary Table S2).

### Party-hubs and date-hubs

For the determination of human party- and date-hubs, human gene expression data were obtained from the Human Protein Atlas^48^, which contains tissue-wise RNA levels (TPM) for 37 tissues, namely the *adipose tissue, adrenal gland, appendix, bone marrow, breast, cerebral cortex, cervix/uterine, colon, duodenum, endometrium, epididymis, esophagus, fallopian tube, gallbladder, heart muscle, kidney, liver, lung, lymph node, ovary, pancreas, parathyroid gland, placenta, prostate, rectum, salivary gland, seminal vesicle, skeletal muscle, skin, small intestine, smooth muscle, spleen, stomach, testis, thyroid gland, tonsil,* and *urinary bladder*. For each interacting protein pair, the RNA levels of both the partners were correlated using the Pearson correlation coefficient (PCC). The mean PCC values for all the partners of the hub proteins were used to classify the hub further into party-hubs and date-hubs^49^. We have used PRISM^50^ webserver to confirm that no two interacting partners of a party hub share the same interacting surface with the latter. The hubs having a mean PCC value ≥ 0.5 were considered as party hubs and those having a PCC value < 0.5 were considered as date hubs^51^. We have also used mean PCC value of all proteins as the cutoff to select party-hubs (above mean) and date-hubs (below mean)^14^.

### Human essential genes

Genes essential for human survival and reproduction, collectively known as essential human genes, were obtained from three recent experiments based on gene trap mutagenesis^52^ and high-resolution CRISPR-screening^53,54^. Human genes (and their encoded proteins) considered as essential or nonessential in all the three screenings were considered as essential and nonessential, respectively. The final data consists of 768 essential and 8080 nonessential human proteins.

### Evolutionary rate

For the calculation of evolutionary rate of human proteins, the nonsynonymous nucleotide substitutions per nonsynonymous site (dN) and synonymous nucleotide substitutions per synonymous site (dS), were obtained from the Ensembl biomart^55^, using 1:1 mouse and chimpanzee orthologs for each human protein. The mutation saturation was controlled by discarding dS values greater than 3 and the dN/dS ratio was used as evolutionary rate^29^.

### Intrinsically disordered proteins

We used IUPred algorithm to predict the intrinsically disordered regions in the protein sequence. In IUPred, each amino acid residue is given a probability score based on its pairwise energy profile with respect to its interaction with other residues along the protein sequence. Residues with scores ≥ 0.50 are considered as disordered and < 0.50 as ordered^34^. We have downloaded the ‘reviewed’ human protein sequence from Uniprot (Accession UP000005640). We discarded all proteins with < 30 amino acid residues. Proteins with a continuous stretch of ≥ 30 disordered residues were considered as proteins with long intrinsically disordered regions. We have calculated the number of these disordered stretches, the proportion of residues in the long-disordered stretches, the total number of disordered amino acid residues and the proportion of disordered amino acid residues for each human protein. Following Panda et al. 2017^56^, human proteins were classified into five groups based on their disorder content: A, Ordered (having 0–20% disordered amino acid residues); B, Moderately disordered (having 20–40% disordered amino acid residues); C, Disordered (having 40–60% disordered amino acid residues); D, Highly disordered (having 60–80% disordered amino acid residues) and E, Extremely disordered (having 80–100% disordered amino acid residues).

### Molecular recognition features

The Molecular recognition features (MoRFs) were obtained from fMoRFpred^35^ webserver. We have selected MoRF regions of ≥ 5 residues and calculated the number of such MoRF regions and total MoRF residues for our study.

### Protein domains

The Ensembl biomart^55^ was used to obtain the interpro^38^ domains of human proteins.

### Functional enrichment analysis

The functional enrichment analysis was carried out using the Gene Ontology^57^ based on Humanmine^39^ and Gorilla^40^ web-servers. The gene ontology terms under different Gene Ontology domains like GO biological process and GO molecular function were used to determine the overrepresented biological processes and molecular functions of pathogen-interacting human proteins. The P-values determining the overrepresented GO terms were corrected using Benjmini-Hochberg correction. The top ten GO biological process and GO molecular function terms represented in both datasets were used as overrepresented GO terms.

### Statistical analyses

All the statistical analyses in this study have been done using in-house PERL script (for Z-test to compare percentages in different samples) and IBM SPSS 22 statistical package (for all other statistical tests)^58^.

## Conclusions

Recent developments of high-throughput interspecific protein–protein interaction data paved the way for host–pathogen interaction studies to understand detailed aspects of pathogenicity, leading to the development of platforms for host-directed therapeutic research. In this study, we explored the attributes of the human–bacteria protein–protein interaction (PPI) network from the available large-scale interspecific interactome data of three bacterial species, *Bacillus anthracis, Francisella tularensis and Yersinia pestis*, for which large-scale high-throughput intraspecific and interspecific PPI data are available. It was observed that the central proteins within intraspecific human and bacterial interactome preferentially participate in human-bacteria interaction. This includes hubs and bottlenecks of both human and bacterial PPI networks. Additionally, within human hubs, party-hubs participate in the interspecific PPI network more often than that of date hubs. It was also revealed that these pathogens preferentially interact with human essential proteins, both within hubs and nonhubs, thereby assisting in disease progression. From evolutionary perspective, these bacterial pathogens interact with evolutionarily more conserved human proteins, leading to a sustainable interaction, helpful for pathogen species. A detailed analysis of host proteins’ structural features revealed that the pathogen-interacting human proteins contain a higher number of protein domains and an abundance of intrinsically disordered residues and regions, which are likely to assist human-bacteria interaction by promoting high-affinity and low-affinity protein–protein interactions, respectively. Furthermore, the functional enrichment in pathogen-interacting human proteins revealed an enrichment of proteins involved in various biological processes, including catalytic functions related to the binding of several biomolecules. These enriched proteins are supposed to regulate essential metabolic and immune system processes, cellular localization, and transport and also influence the binding of host macromolecules and cell-adhesion molecules that are necessary for host-microbial cross-talks.

## Supplementary Information

Supplementary Information.

## Data Availability

All the data are available upon request.

## Abbreviations

PPI: Protein–protein interaction
PHPPI: Pathogen–host protein–protein interaction
GO: Gene ontology
dN: Nonsynonymous nucleotide substitutions per nonsynonymous site
dS: Synonymous nucleotide substitutions per synonymous site

## References

[1] Durmus Tekir S, Cakir T, Ulgen K (2012). Infection strategies of bacterial and viral pathogens through pathogen–human protein–protein interactions. Front. Microbiol..

[2] Ahmed H (2018). Network biology discovers pathogen contact points in host protein–protein interactomes. Nat. Commun..

[3] Saha S, Sengupta K, Chatterjee P, Basu S, Nasipuri M (2017). Analysis of protein targets in pathogen–host interaction in infectious diseases: A case study on *Plasmodium falciparum* and Homo sapiens interaction network. Brief. Funct. Genom..

[4] Bahia D, Satoskar AR, Dussurget O (2018). Cell signaling in host–pathogen interactions: The host point of view. Front. Immunol..

[5] Blasi F, Tarsia P, Aliberti S (2005). Strategic targets of essential host–pathogen interactions. Respiration.

[6] Neu HC (1992). The crisis in antibiotic resistance. Science.

[7] Nicod C, Banaei-Esfahani A, Collins BC (2017). Elucidation of host–pathogen protein–protein interactions to uncover mechanisms of host cell rewiring. Curr. Opin. Microbiol..

[8] Halehalli RR, Nagarajaram HA (2014). Molecular principles of human virus protein–protein interactions. Bioinformatics.

[9] Schleker S, Trilling M (2013). Data-warehousing of protein–protein interactions indicates that pathogens preferentially target hub and bottleneck proteins. Front. Microbiol..

[10] He X, Zhang J (2006). Why do hubs tend to be essential in protein networks?. PLoS Genet..

[11] Hahn MW, Kern AD (2005). Comparative genomics of centrality and essentiality in three eukaryotic protein-interaction networks. Mol. Biol. Evol..

[12] Jeong H, Mason SP, Barabasi AL, Oltvai ZN (2001). Lethality and centrality in protein networks. Nature.

[13] Tew KL, Li X-L, Tan S-H (2007). Functional centrality: Detecting lethality of proteins in protein interaction networks. Genome Inform..

[14] Ekman D, Light S, Björklund ÅK, Elofsson A (2006). What properties characterize the hub proteins of the protein–protein interaction network of *Saccharomyces cerevisiae*?. Genome Biol..

[15] Fraser HB, Hirsh AE, Steinmetz LM, Scharfe C, Feldman MW (2002). Evolutionary rate in the protein interaction network. Science.

[16] Helsen J, Frickel J, Jelier R, Verstrepen KJ (2019). Network hubs affect evolvability. PLoS Biol..

[17] Alvarez-Ponce D, Feyertag F, Chakraborty S (2017). Position matters: Network centrality considerably impacts rates of protein evolution in the human protein–protein interaction network. Genome Biol. Evol..

[18] Becerra A, Bucheli VA, Moreno PA (2017). Prediction of virus-host protein–protein interactions mediated by short linear motifs. BMC Bioinform..

[19] García-Pérez CA, Guo X, Navarro JG, Aguilar DAG, Lara-Ramírez EE (2018). Proteome-wide analysis of human motif-domain interactions mapped on influenza A virus. BMC Bioinform..

[20] Yang H (2011). Insight into bacterial virulence mechanisms against host immune response via the Yersinia pestis-human protein–protein interaction network. Infect. Immun..

[21] Stark C (2006). BioGRID: A general repository for interaction datasets. Nucleic Acids Res..

[22] Chatr-Aryamontri A (2006). MINT: The molecular INTeraction database. Nucleic Acids Res..

[23] Peri S (2004). Human protein reference database as a discovery resource for proteomics. Nucleic Acids Res..

[24] Xenarios I (2002). DIP, the database of interacting proteins: A research tool for studying cellular networks of protein interactions. Nucleic Acids Res..

[25] Hermjakob H (2004). IntAct: An open source molecular interaction database. Nucleic Acids Res..

[26] Liao B-Y, Scott NM, Zhang J (2006). Impacts of gene essentiality, expression pattern, and gene compactness on the evolutionary rate of mammalian proteins. Mol. Biol. Evol..

[27] Acharya D, Mukherjee D, Podder S, Ghosh TC (2015). Investigating different duplication pattern of essential genes in mouse and human. PLoS ONE.

[28] Chen H (2019). New insights on human essential genes based on integrated analysis and the construction of the HEGIAP web-based platform. Brief. Bioinform..

[29] Acharya D, Ghosh TC (2016). Global analysis of human duplicated genes reveals the relative importance of whole-genome duplicates originated in the early vertebrate evolution. BMC Genom..

[30] Mészáros B, Simon I, Dosztányi Z (2009). Prediction of protein binding regions in disordered proteins. PLoS Comput. Biol..

[31] Dunker AK, Cortese MS, Romero P, Iakoucheva LM, Uversky VN (2005). Flexible nets: The roles of intrinsic disorder in protein interaction networks. FEBS J..

[32] Dunker AK, Romero P, Obradovic Z, Garner EC, Brown CJ (2000). Intrinsic protein disorder in complete genomes. Genome Inform..

[33] Ward JJ, Sodhi JS, McGuffin LJ, Buxton BF, Jones DT (2004). Prediction and functional analysis of native disorder in proteins from the three kingdoms of life. J. Mol. Biol..

[34] Dosztanyi Z, Csizmok V, Tompa P, Simon I (2005). IUPred: Web server for the prediction of intrinsically unstructured regions of proteins based on estimated energy content. Bioinformatics.

[35] Disfani FM (2012). MoRFpred, a computational tool for sequence-based prediction and characterization of short disorder-to-order transitioning binding regions in proteins. Bioinformatics.

[36] Uversky VN, Oldfield CJ, Dunker AK (2008). Intrinsically disordered proteins in human diseases: Introducing the D2 concept. Annu. Rev. Biophys..

[37] Basu MK, Poliakov E, Rogozin IB (2009). Domain mobility in proteins: Functional and evolutionary implications. Brief. Bioinform..

[38] Hunter S (2008). InterPro: The integrative protein signature database. Nucleic Acids Res..

[39] Smith RN (2012). InterMine: A flexible data warehouse system for the integration and analysis of heterogeneous biological data. Bioinformatics.

[40] Eden E, Navon R, Steinfeld I, Lipson D, Yakhini Z (2009). GOrilla: A tool for discovery and visualization of enriched GO terms in ranked gene lists. BMC Bioinform..

[41] Prieto C, De Las Rivas J (2006). APID: Agile protein interaction DataAnalyzer. Nucleic Acids Res..

[42] Calderone A, Castagnoli L, Cesareni G (2013). Mentha: A resource for browsing integrated protein-interaction networks. Nat. Methods.

[43] Ammari MG, Gresham CR, McCarthy FM, Nanduri B (2016). HPIDB 2.0: A curated database for host–pathogen interactions. Database.

[44] Durmuş Tekir S (2013). PHISTO: Pathogen–host interaction search tool. Bioinformatics.

[45] Gioutlakis A, Klapa MI, Moschonas NK (2017). PICKLE 2.0: A human protein–protein interaction meta-database employing data integration via genetic information ontology. PLoS ONE.

[46] Szklarczyk, D. *et al.* The STRING database in 2017: Quality-controlled protein–protein association networks, made broadly accessible. *Nucleic Acids Res.* gkw937 (2016). 10.1093/nar/gkw937 PMC5210637 27924014

[47] Newman MEJ (2005). Power laws, Pareto distributions and Zipf's law. Contemp. Phys..

[48] Uhlen M (2010). Towards a knowledge-based human protein atlas. Nat. Biotechnol..

[49] Han J-DJ (2004). Evidence for dynamically organized modularity in the yeast protein–protein interaction network. Nature.

[50] Baspinar A, Cukuroglu E, Nussinov R, Keskin O, Gursoy A (2014). PRISM: A web server and repository for prediction of protein–protein interactions and modeling their 3D complexes. Nucleic Acids Res..

[51] Batada NN (2007). Still stratus not altocumulus: Further evidence against the date/party hub distinction. PLoS Biol..

[52] Blomen VA (2015). Gene essentiality and synthetic lethality in haploid human cells. Science.

[53] Wang T (2015). Identification and characterization of essential genes in the human genome. Science.

[54] Hart T (2015). High-resolution CRISPR screens reveal fitness genes and genotype-specific cancer liabilities. Cell.

[55] Yates A (2016). Ensembl 2016. Nucleic Acids Res..

[56] Panda A, Acharya D, Ghosh TC (2017). Insights into human intrinsically disordered proteins from their gene expression profile. Mol. BioSyst..

[57] Gene Ontology C (2004). The Gene Ontology (GO) database and informatics resource. Nucleic Acids Res..

[58] Nie NH, Bent DH, Hull CH (1970). SPSS: Statistical Package for the Social Sciences.

