# Supplementary material for: Elucidating the network features and evolutionary attributes of intra- and interspecific protein–protein interactions between human and pathogenic bacteria
Source: Sci Rep. 2021 Jan 8;11:190. doi: 10.1038/s41598-020-80549-x (PMC7794237; doi:10.1038/s41598-020-80549-x)
Supplement: Supplementary file 1 — Supplementary Information. [file 41598_2020_80549_MOESM1_ESM.pdf]

Supporting Information

Elucidating the Network Features and Evolutionary Attributes of Intra- and Interspecific Protein–Protein Interactions between Human and Pathogenic Bacteria

Authors:

Debarun Acharya (debarunacharya@gmail.com)

Tapan K. Dutta* (tapan@jcbose.ac.in)

Affiliation:

Department of Microbiology,
Bose Institute,
P-1/12, CIT Scheme VII M,
Kolkata- 700 054, West Bengal
India
Phone/fax: +91 33 2569 3241.

* Corresponding Author.

Supplementary Figure

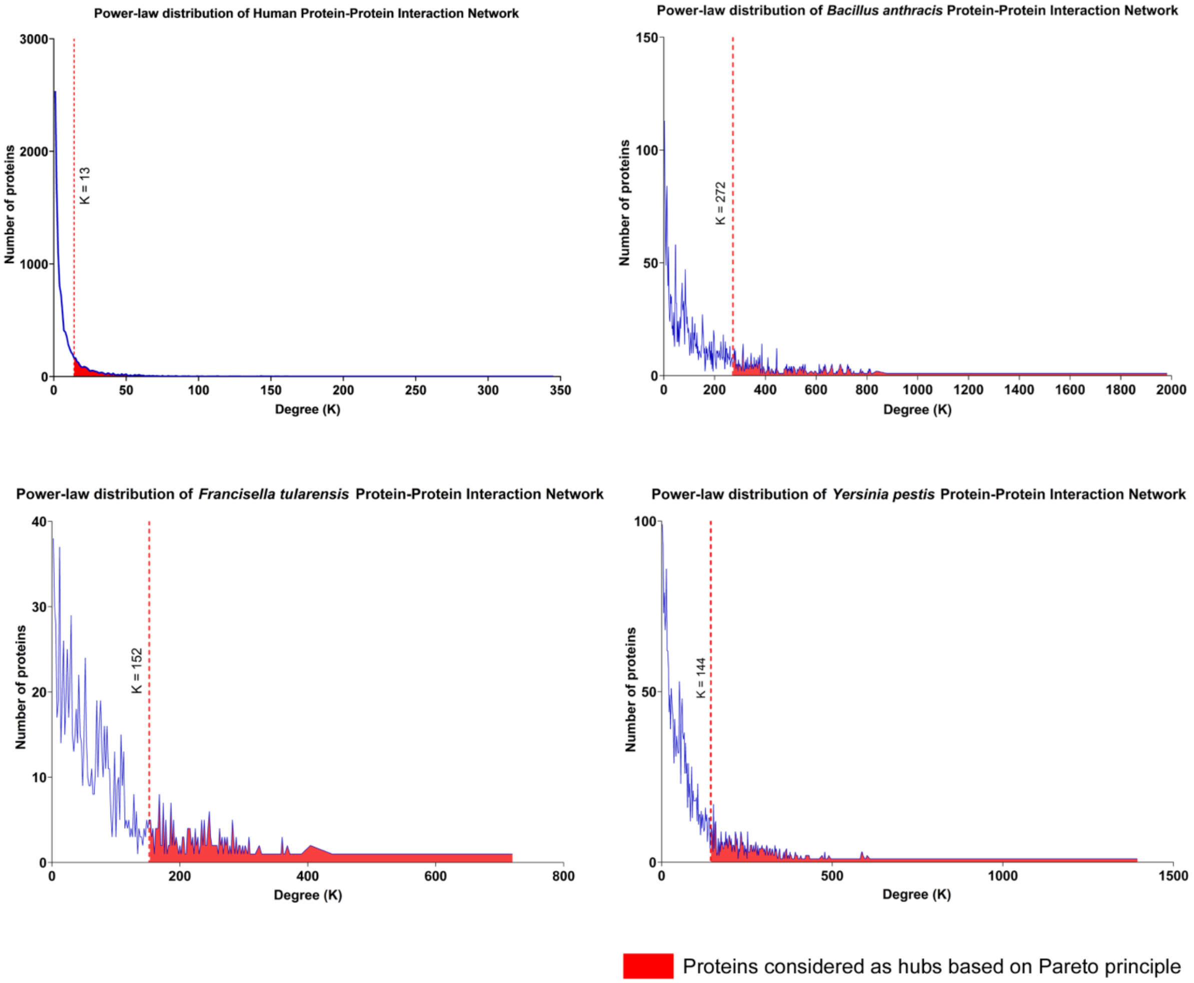

Figure S1. Node degree distribution of human proteome and bacterial proteome. The Pareto principle was applied to obtain the hubs (represented in red colour) as the top 20% nodes of the power-law node degree distribution. K values represent the threshold of node degree for each species.

Supplementary Tables

Table S1. Human-pathogen protein-protein interactions used in this study.

Pathogen	Database				Interactions supported by at least three databases
	APID	MENTHA	HPI-DB	PHISTO	
Bacillus anthracis	3015	3006	3019	3109	2969
Francisella tularensis	1307	1307	1330	1352	1305
Yersinia pestis	3922	3923	3924	4103	3919

Table S2. The number of hubs and bottlenecks in bacterial protein-protein interaction network.

Bacterial species	Experimentally verified interactions	Hubs	Bottlenecks	Total (N)
Bacillus anthracis	277210	659	657	3285
Francisella tularensis	53614	236	234	1167
Yersinia pestis	135090	575	576	2872

Table S3. Proportion of hubs and bottlenecks in pathogenic bacteria-interacting and non-interacting human proteins.

Proportion of hubs in pathogen-interacting and non-interacting human proteins					
Pathogen interaction status	Total Proteins	Hubs	%Hubs	Mean Interacting partners	Significance
Non-interacting	9137	1498	16.39	8.95	Z = 16.265, P < 0.01, two-tailed Z-test
Interacting	2678	819	30.58	15.06	
Proportion of bottlenecks in pathogen-interacting and non-interacting human proteins					
Pathogen interaction status	Total Proteins	Bottlenecks	%Bottlenecks	Significance	
Non-interacting	9137	1540	16.85	Z = 15.557, P < 0.01, two-tailed Z-test	
Interacting	2678	817	30.51		

Table S4. Disorder status of pathogenic bacteria-interacting and non-interacting human proteins.

Pathogen-interaction status	Mean number of long disordered regions	Mean percentage of long disordered region	Mean number of short disordered regions	Mean percentage of short disordered region	Mean number of total disordered residues	Mean percentage of disordered residues
Pathogen interacting	1.824	18.888	3.232	22.923	237.486	28.410
Pathogen noninteracting	1.187	15.067	2.164	18.820	151.616	23.881
P-value (Mann-Whitney U test)	3.93×10^{-37}	1.76×10^{-22}	4.14×10^{-39}	8.26×10^{-21}	1.18×10^{-47}	6.67×10^{-21}

Table S5. Correlation of human protein intrinsic disorder with the number of interacting pathogenic bacterial proteins.

Bacterial pathogen	Number of long disordered regions in human proteins	Proportion of long disordered regions in the protein sequence	Number of all disordered regions in human proteins	Proportion of all disordered residues in human protein sequence
Bacillus anthracis	0.092	0.071	0.107	0.071
Francisella tularensis	0.067	0.051	0.079	0.055
Yersinia pestis	0.094	0.070	0.106	0.067
All correlations are significant at 0.01 level (2-tailed).				

Table S6. Enriched Gene Ontology terms of bacterial pathogen-interacting human proteins for Biological Process and Molecular Function.

Gene Ontology	P-value (Humanmine)	P-value (GOrilla)
Gene Ontology (Biological Process)		
Regulation of biological process (GO:0050789)	4.806×10^{-56}	1.71×10^{-18}
Cellular localization (GO:0051641)	6.054×10^{-44}	8.35×10^{-16}
Immune system process (GO:0002376)	2.477×10^{-45}	2.91×10^{-25}
Interspecies interaction between organisms (GO:0044419)	2.563×10^{-37}	3.64×10^{-17}
Regulation of cellular metabolic process (GO:0031323)	1.920×10^{-44}	1.18×10^{-16}
Regulation of cellular process (GO:0050794)	1.467×10^{-38}	2.22×10^{-19}
Regulation of metabolic process (GO:0019222)	6.943×10^{-45}	4.07×10^{-16}
Regulation of nitrogen compound metabolic process (GO:0051171)	5.535×10^{-46}	1.27×10^{-15}
Regulation of primary metabolic process (GO:0080090)	4.861×10^{-40}	4.53×10^{-16}
Vesicle-mediated transport (GO:0016192)	8.807×10^{-39}	5.67×10^{-24}
Gene Ontology (Molecular Function)		
RNA binding (GO:0003723)	9.386×10^{-40}	5.82×10^{-26}
enzyme binding (GO:0019899)	6.178×10^{-48}	6.46×10^{-21}
Nucleic acid binding (GO:0003676)	5.314×10^{-23}	1.42×10^{-18}
Protein binding (GO:0005515)	9.599×10^{-146}	2.79×10^{-15}
Protein-containing complex binding (GO:0044877)	1.509×10^{-21}	9.64×10^{-15}
Cadherin binding (GO:0045296)	1.387×10^{-20}	8.21×10^{-14}
Cell adhesion molecule binding (GO:0050839)	8.513×10^{-20}	1.01×10^{-12}
Transcription factor binding (GO:0008134)	7.195×10^{-18}	3.27×10^{-9}
Chromatin binding (GO:0003682)	8.058×10^{-11}	8.30×10^{-9}
Kinase binding (GO:0019900)	1.701×10^{-16}	2.81×10^{-8}
